# Protrusion-Mediated Signaling Regulates Patterning of the Developing Nervous System

**DOI:** 10.3389/fcell.2020.579073

**Published:** 2020-09-29

**Authors:** Rachel E. Moore, Jon Clarke, Paula Alexandre

**Affiliations:** ^1^Department of Developmental Neurobiology, King’s College London, London, United Kingdom; ^2^Developmental Biology and Cancer, UCL GOS Institute of Child Health, University College London, London, United Kingdom

**Keywords:** neuronal patterning, neuronal spacing, protrusion mediated signaling, long distance signaling, nervous system, neurogenesis

## Abstract

During brain development, the tissue pattern and specification are the foundation of neuronal circuit formation. Contact-mediated lateral inhibition is well known to play an important role in determining cell fate decisions in the nervous system by either regulating tissue boundary formation or the classical salt-and-pepper pattern of differentiation that results from direct neighboring cell contacts. In many systems, however, such as the *Drosophila* notum, *Drosophila* wing, zebrafish pigmented cells, and zebrafish spinal cord, the differentiation pattern occurs at multiple-cell diameter distances. In this review, we discuss the evidence and characteristics of long-distance patterning mechanisms mediated by cellular protrusions. In the nervous system, cellular protrusions deliver the Notch ligand Delta at long range to prevent cells from differentiating in their vicinity. By temporal control of protrusive activity, this mechanism can pattern differentiation in both space and time.

## Introduction

During morphogenesis, the differentiation of cells must be coordinated and patterned at both short (among immediate neighbors) and long range (across several or many-cell diameters). Short-range signaling can be achieved, for example, by cell–cell contact via ligands and receptors proteins inserted into cell membranes (such as Delta-Notch or ephrin-Eph signaling) (reviewed by Cayuso et al., 2015; Henrique and Schweisguth, 2019). Long-range signaling requires mechanisms that can operate over greater distances and is traditionally thought to employ secreted ligands [for example, hedgehog (Hh), wingless (Wnt), fibroblast growth factor (FGF), or bone morphogenic protein (BMP)] that diffuse through tissues to their distant target receiving cells (Briscoe and Small, 2015). More recently, it has become apparent that morphogen and cell-to-cell contact-dependent signaling can also be achieved between distant cells via long cellular protrusions (for example, Cohen et al., 2010; Eom et al., 2015; Osswald et al., 2015; and reviewed by González-Méndez et al., 2019). Cellular protrusions that may have signaling, organizational, or mechanical roles have been described in many systems and can have a variety of morphologies, cytoskeletal structure, and names (reviewed by Kornberg, 2014). Here, we will focus on protrusions called cytonemes, nanotubes, and filopodia that include actin-based projections, which together with more substantial protrusions can contain both microtubule and actin cytoskeletons. In this discussion, we will concentrate on protrusion-mediated signals in the nervous system (Table 1). Of course, in the nervous system, the most obvious effectors of long-distance communication via cell protrusions are the axons and dendrites that mediate electrical and chemical transmission often over exceptionally long distances, but we will not deal with this here. Other protrusions from neuronal precursors set up long-range pattern and coordinate neurodevelopmental events. The cell protrusions in protrusion-mediated signaling can either deliver ligand over long distances and/or can act as sensors that receive signals by reaching out and capturing distant ligands (reviewed by González-Méndez et al., 2019). Here we begin by discussing protrusion-mediated delivery of signals in the developing and adult nervous system, and then we focus on recent work in the vertebrate spinal cord that shows protrusions can control both long-distance spatial and temporal patterns of neuronal differentiation.

**TABLE 1:** Summary of protrusion mediated signaling in the nervous system.

Organism/cell type	Protrusion type	Structural components	Length and lifetime	Known cargo/signaling pathway	Function
Zebrafish embryo/neural plate cells (Stanganello et al., 2015)	Cytoneme – multidirectional	Actin Tubulin is present at the base	10–50 μm Those carrying Wnt8a measure 16.6 μm on average.	Wnt8a	Mediate long-range Wnt signaling and pattern the neural plate
Zebrafish embryo/neuronal precursors in the spinal cord (Hadjivasiliou et al., 2019)	Basal protrusion – bidirectional along the A/P axis	Microtubules (Hadjivasiliou et al., 2019)	Average 42.6 μm length (4-cell diameters) Remain elongated for 6.8 h on average	Delta	Mediate long-distance Delta Notch signaling pathway activation – pattern neuronal differentiation along the zebrafish spinal cord
*Drosophila* notum/SOP cells (De Joussineau et al., 2003; Cohen et al., 2010)	Basal filopodia – multidirectional	Actin	Filopodia formed by small bristles precursors (microchaetes) measure on average 11 μm (spanning 1.4-cell diameters) and last <10 min, while in macrochaetes filopodia can span 120 μm (12- to 21-cell diameters)	Delta	Mediate long-distance Delta-Notch signaling pathway activation – pattern mechanosensory bristles precursors in *Drosophila* notum
Adult zebrafish brain/neural stem cells and progenitors (Obermann et al., 2019)	Apical and basal filopodia-like protrusions – multidirectional	Some filopodia have F-actin	The longest filopodia span 4-cell diameters	Delta	Unknown
Rodent cortex/intermediate progenitors (Noctor et al., 2004; Nelson et al., 2013)	Long and short cellular protrusions. Long protrusion is directed toward the apical surface while short protrusions are multidirectional	Unknown	Unknown	Delta	Suggested to mediate long-distance Delta-Notch signaling pathway activation and maintain radial glia cells in proliferation
Neuronal cocultures (reviewed in Victoria and Zurzolo, 2017)	Tunneling nanotubes	May contain microtubules or F-actin	Up to 100 μm	α-Synuclein, amyloid-β, huntingtin, tau, and prion	Transport components that have been associated with neurodegenerative diseases. May mediate the propagation of disease components to healthy cells or healthy components to diseased cells
Rat/hippocampal neurons cocultured with astrocytes (Wang et al., 2012)	Tunneling nanotubes – directed toward astrocytes	Contain microtubules and some also contain F-actin	Up to 30-μm length and 15-min lifetime	Can contain connexin43	Regulate electrical coupling between immature neurons and astrocytes

## Cytonemes and Nanotubes in the Developing and Adult Nervous System

Cytonemes are actin-rich membranous tubes of less than 1-μm diameter and can be up to several-hundred microns in length, with some containing tubulin at the base (González-Méndez et al., 2019). The first evidence that cell–cell signaling might be mediated by cytonemes in the developing vertebrate nervous system was the observation that fluorescently tagged Wnt8a protein localizes to and can be released from the tips of cytonemes protruding from cells in the very early zebrafish neural plate (Luz et al., 2014). Shortly after this, it was shown these cytonemes not only contact receiving cells and activate Wnt signaling, but also that experimental regulation of cytoneme length can alter the signaling range of the Wnt ligand and thus modify regional patterning in the neural plate (Stanganello et al., 2015). Interestingly, the Wnt producing cells regulate their own cytoneme production via an autocrine Wnt signal that activates the Ror2 receptor and the planar cell polarity pathway downstream (Mattes et al., 2018).

In adult brain, glioblastoma cells were recently shown to develop protrusions with cytoneme-like identity called tumor microtubes. Patient-derived gliomas seeded into a mouse brain use tumor microtubes for invasion and proliferation and form interconnections over long distances (Osswald et al., 2015). In a *Drosophila* model of glioma, tumor microtubes enwrap neurons and deplete the neurons of Wnt while activating Wnt signaling in the tumor cells. Tumor microtubes thus lead to neurodegeneration and tumor progression (Portela et al., 2019).

Another distinct type of thin cellular protrusion has also been implicated in long-range communication in embryo development and in normal and diseased adult brains. These are tunneling open-ended nanotubes, similar to cytonemes in that they are very narrow and membranous, with varied cytoskeleton composition, but distinct from cytonemes in that they fuse with their targets to form cytoplasmic continuity. Nanotube connections are capable of electrical coupling and delivering cytoplasmic contents including small organelles between distant cells (Wang et al., 2012; Gerdes et al., 2013). In the adult nervous system, tunneling nanotubes have been proposed to distribute mediators of neurodegenerative disease such as α-synuclein, amyloid-β, huntingtin, tau, and prions (reviewed in Victoria and Zurzolo, 2017).

## Larger Cellular Protrusions in Neuronal Development

In the *Drosophila* notum, differentiating sensory organ precursor (SOP) cells use basal protrusions and filopodia to organize a mosaic pattern of differentiation spaced on average 4.6-cell diameters apart. In microchaete precursors, these cell extensions, visualized by CD8-GFP or Moe-GFP expression, appear as filopodia (Cohen et al., 2010), whereas macrochaete precursors develop a mixture of filopodia and larger protrusions (De Joussineau et al., 2003). These protrusions can span 1.4- to 21-cell diameters in length (11–120 μm) and are highly dynamic. Differentiating SOP cells and their respective filopodia express membrane bound Delta (De Joussineau et al., 2003; Cohen et al., 2010) and are able to activate Notch signaling to prevent neighboring and more distant cells from differentiating (Cohen et al., 2010). Ablation of a differentiating SOP switches on the expression of SOP-specific genes in neighboring cells to replace it, suggesting the differentiating SOPs are inhibiting their neighbors from differentiating. Reducing filopodia length or Delta-Notch signaling leads to a decrease in the spacing between SOP cells. These studies support the view that long-distance Delta-Notch–based lateral inhibition is delivered by filopodial protrusions and provide a mechanism by which the sparse induction of SOP cells can be generated (Cohen et al., 2010).

Less understood is the function of long protrusions and filopodia-like structures reported in the rodent cortex and adult zebrafish brain (Noctor et al., 2004; Chapouton et al., 2010; Nelson et al., 2013; Obermann et al., 2019). In the rodent brain, intermediate progenitors (that undergo mitosis in non-apical locations) were described as expressing Delta and having short- and long-range protrusions and a large number of multidirectional membrane protrusions that contact radial glia processes (Nelson et al., 2013). In adult zebrafish, both neural stem cells (NSCs) and neural progenitors also develop multiple multidirectional filopodia-like actin-enriched structures. The activated NSC and neural progenitors express Delta (Chapouton et al., 2010). This raises the possibility that a similar cell protrusion-mediated mechanism may also exist in rodents and adult zebrafish brains to deliver Delta-Notch lateral inhibition at a distance of several-cell diameters. However, the dynamics and pattern of differentiation surrounding the intermediate progenitors or activated NSCs or neural progenitors has never been determined and therefore the protrusions’ function in these cases remains largely unknown.

**FIGURE 1:**
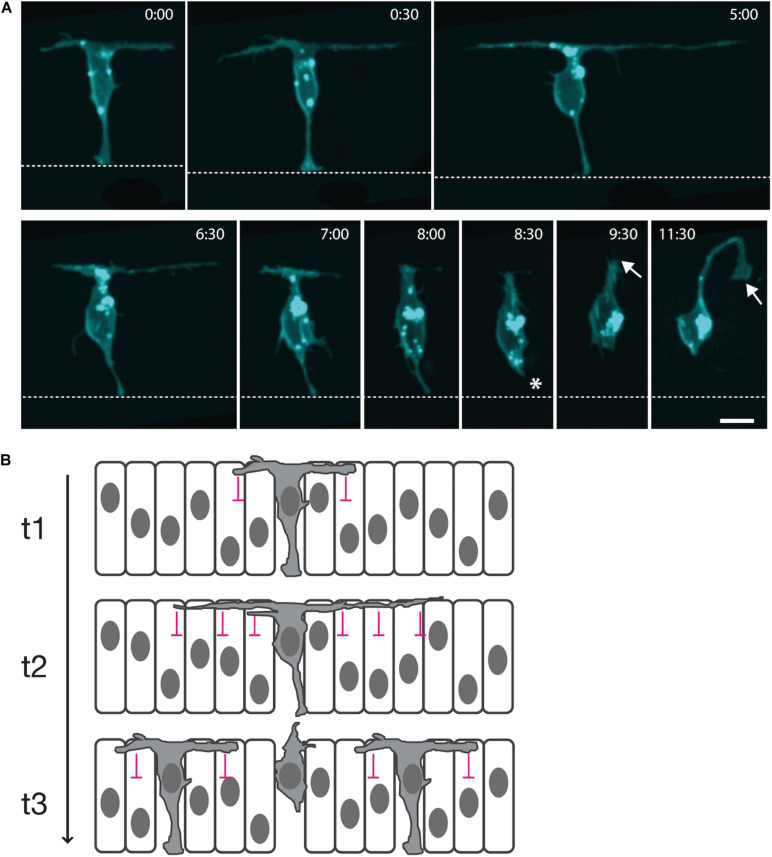
Long cellular protrusions play a role in the spatiotemporal patterning of zebrafish spinal neuron differentiation. **(A)** Live *in vivo* imaging in the zebrafish embryonic spinal cord of a single neuron labeled with a membrane marker. The cell body positions to the basal surface of the neuroepithelium while maintaining an attachment to the apical surface (dashed line; 0:00). The neuron extends two long protrusions along the basal surface, one anteriorly and one posteriorly (0:00–5:00). Each basal protrusion spans several-cell diameters. Both are retracted into the cell body (6:30–8:00), along with the apical attachment (asterisk; 8:30), before the neuron extends an axon (arrow; 9:30–11:30). **(B)** Diagrammatic working model of transient long-distance lateral inhibition delivered via basal protrusions. t1: A differentiating neuron expresses Delta (gray cytoplasm) and begins to extend basal protrusions. Delta signaling from the basal protrusions induces Notch signaling in the neighboring neuroepithelial cells that they contact, inhibiting their neuronal differentiation (lateral inhibition delivered by basal protrusions is represented by pink signs). t2: The basal protrusions grow to span several-cell diameters and inhibit the neuronal differentiation of neuroepithelial progenitors at a distance. t3: Retraction of the basal protrusions occurs before axon initiation, releasing the neuroepithelial cells that receive least contact with basal protrusions to differentiate.

Recently, long transient protrusions have been shown to mediate long-distance spatiotemporal patterning of spinal neurons in vertebrates (Hadjivasiliou et al., 2019). Previous work had established that neurons of any particular subtype initially differentiate along the spinal cord in a sparse pattern with gaps of several-cell diameters between them (Dale et al., 1987; Roberts et al., 1987; Higashijima S. et al., 2004a; Higashijima S.-I. et al., 2004b; Batista et al., 2008). Subsequent neurons then arise in these gaps to eventually produce a continuous column of neurons of the same subtype. Live *in vivo* imaging of newly differentiating spinal neurons in the zebrafish embryo was used to uncover the mechanism of this spatiotemporal pattern. When spinal neuron cell bodies reach the basal surface of the neuroepithelium, they extend two long protrusions at the basal surface of the neuroepithelia, one anteriorly and one posteriorly, which span several-cell diameters (Hadjivasiliou et al., 2019). These protrusions have strict directionality; they last for several hours, and they are microtubule-based. Basal protrusions are then fully retracted into the cell body, and at the same time, the neuron detaches from the apical surface and before it extends an axon and dendrites (Figure 1A). This protrusive behavior is also fully replicated by spinal non-apical progenitors (which divide terminally to generate two neurons) while undergoing apical detachment (McIntosh et al., 2017; Hadjivasiliou et al., 2019).

The timing and morphology of basal protrusions hinted that they may play a role in the spatiotemporal patterning of spinal cord neuronal differentiation. The mean length of each basal protrusion is just over 40 μm, and about 90% of contemporary differentiation events occur outside the reach of these basal protrusions. However, later differentiation events did occur within this distance. As such, there is a negative correlation between the distance between two cells and the time at which they differentiate, so that cells that are closer together in space tend to differentiate further apart in time and vice versa.

It had previously been shown that Delta expression is required for the sparse spatial patterning of zebrafish spinal cord neurons (Okigawa et al., 2014), and DeltaD protein is specifically enriched in basal protrusions, while a transgenic Notch signaling reporter is upregulated in cells within their reach (Hadjivasiliou et al., 2019). This suggested that DeltaD signaling from basal protrusions could promote Notch signaling in long-distance neighbors and so delay their differentiation (Figure 1B).

This hypothesis was interrogated further using a combination of experimental and mathematical approaches. Basal protrusion length is significantly reduced in the absence of the extracellular matrix protein laminin, and this correlated with a reduction in the distance between neurons differentiating close together in time. Mathematical modeling built on previous models of Delta-Notch signaling dynamics (Collier et al., 1996; Cohen et al., 2010) first confirmed that spatiotemporal patterns of differentiation *in vivo* are unlikely to be randomly generated. Further simulations that incorporated experimentally observed protrusion dynamics from wild-type and laminin-deficient zebrafish embryos then showed that the spatiotemporal dynamics of differentiation in both wild-type and laminin-deficient embryos can be explained by lateral inhibition mediated by basal protrusions. Importantly, the mathematical model predicts that the experimental differences in neuronal patterning observed between wild-type and laminin-deficient embryos can be explained by the differences in the length of their basal protrusions. Finally, the mathematical model strongly suggests that only Delta-Notch signaling via basal protrusions can recapitulate the *in vivo* spatiotemporal patterning of neuronal differentiation. Including soma-to-soma lateral inhibition (either with or without basal protrusion signaling) leads to patterning that does not match *in vivo* observations (Hadjivasiliou et al., 2019). This is consistent with basal protrusions being the main mechanism that regulates both the position and timing of spinal cord neuron differentiation. We speculate that controlling the timing and position of neuronal differentiation in the spinal cord may be important for neuronal circuit formation, by allowing only a certain number of neurons to join or form a circuit at a certain time. Importantly, these studies, together with those on the pattern of SOPs on the fly’s notum, show that similar protrusion-mediated lateral inhibition mechanisms occur in diverse nervous systems, suggesting similar long-distance lateral inhibition mechanisms may pattern cell differentiation in many nervous systems.

## Conclusion

In this brief article, we summarize the evidence that a variety of different cellular protrusions can mediate long-distance signaling to control tissue patterning or long-distance communication. By focusing on the evolutionarily diverse systems that generate the sparse pattern of SOP differentiation in the fly notum and the spatiotemporal pattern of spinal neuron differentiation in the vertebrate spinal cord, we suggest protrusion-mediated Delta-Notch signaling may be a widespread mechanism of spatial patterning in the nervous system.

Long-distance patterning in the nervous system and elsewhere can also be achieved through diffusion of ligands in the manner of the classic morphogen hypothesis. So what might be the advantage of protrusion-mediated signaling? Two possibilities could be considered. One is that protrusions introduce the possibility of precisely controlling the directionality and range of the signal. In the case of the basal protrusions on newly differentiating spinal neurons, the main branches of the protrusions are strictly directed along the anterior and posterior axes (Hadjivasiliou et al., 2019). Although secondary smaller twigs may deliver signals in other directions, the main branches will clearly bias the extended range of signals, along particular anteroposterior channels. The finite length and transient nature of the protrusions additionally limit the range of the signal in time and space.

A second potential advantage is that protrusions offer the possibility of adding selectivity among the cells targeted to receive the signal. Thus, if the target region is a heterogeneous group of cells, cell recognition signals could specify which cells within range to connect with and which to avoid. In some systems, the signals transported by the cytoneme (ligand, receptors, or both) are specific to the type of protrusion and are also responsible for their formation (Roy et al., 2011; Du et al., 2018). The specific interactions between protrusions and target cells clearly depend on the presence of relevant ligands and receptors, but how specificity of interactions is achieved is not entirely clear. In the case of the differentiating spinal neurons, the basal protrusions may potentially contact neural progenitors with different dorsoventral specifications, but additional molecular recognition signals could restrict the delivery of signals to progenitors of a particular dorsoventral identity and thus regulate spatiotemporal pattern in a specific neuronal subtype. There is increasing evidence that signaling through cytonemes requires synaptic components (Huang et al., 2019; Junyent et al., 2020). For example, cytoneme-mediated Wnt signaling between trophoblast stem (TS) cells and embryonic stem (ES) cells was found to be stabilized by the development of synapse-like contacts between the ES cell cytonemes and the Wnt ligand expressing TS cells. The results show cytoneme contacts can select between different Wnt ligands and suggest stabilized cytoneme contacts depended on glutamate receptor–mediated Ca transients (Junyent et al., 2020). This and other work (Kornberg and Roy, 2014) raise the possibility that protrusion-mediated lateral inhibition in the fly and fish nervous system might also use synapse-like contacts to enable cell-specific signaling.

## Author Contributions

JC, PA, and REM wrote the manuscript and prepared the Figure 1. All authors contributed to the article and approved the submitted version.

## Conflict of Interest

The authors declare that the research was conducted in the absence of any commercial or financial relationships that could be construed as a potential conflict of interest.
